# Interventions in Small Food Stores to Change the Food Environment, Improve Diet, and Reduce Risk of Chronic Disease

**Published:** 2012-02-16

**Authors:** Joel Gittelsohn, Megan Rowan, Preety Gadhoke

**Affiliations:** International Health, Johns Hopkins Bloomberg School of Public Health; Johns Hopkins Bloomberg School of Public Health, Baltimore, Maryland; Johns Hopkins Bloomberg School of Public Health, Baltimore, Maryland

## Abstract

**Introduction:**

Many small-store intervention trials have been conducted in the United States and other countries to improve the food environment and dietary behaviors associated with chronic disease risk. However, no systematic reviews of the methods and outcomes of these trials have been published. The objective of this study was to identify small-store interventions and to determine their impact on food availability, dietary behaviors, and psychosocial factors that influence chronic disease risk.

**Methods:**

From May 2009 through September 2010, we used PubMed, web-based searches, and listservs to identify small-store interventions that met the following criteria: 1) a focus on small food stores, 2) a completed impact evaluation, and 3) English-written documentation (peer-reviewed articles or other trial documents). We initially identified 28 trials; 16 met inclusion criteria and were used for analysis. We conducted interviews with project staff to obtain additional information. Reviewers extracted and reported data in a table format to ensure comparability between data.

**Results:**

Reviewed trials were implemented in rural and urban settings in 6 countries and primarily targeted low-income racial/ethnic minority populations. Common intervention strategies included increasing the availability of healthier foods (particularly produce), point-of-purchase promotions (shelf labels, posters), and community engagement. Less common strategies included business training and nutrition education. We found significant effects for increased availability of healthy foods, improved sales of healthy foods, and improved consumer knowledge and dietary behaviors.

**Conclusion:**

Trial impact appeared to be linked to the increased provision of both healthy foods (supply) and health communications designed to increase consumption (demand).

## Introduction

Small food stores, which are common in low-income areas with a high proportion of racial/ethnic minorities (1-8), often have limited healthy options (5-12) and are associated with overconsumption of high-fat, high-sugar foods (11-15) and high rates of obesity and chronic disease (16-20). In recent years, public health practitioners have aimed to improve the food environment and purchasing patterns in small food stores (21-24), yet studies summarizing these interventions and their effectiveness are lacking.

Our objective was to identify small-store intervention strategies that produce significant increases in healthy food access and consumption. Specifically, we sought to present the design and evaluation components of each trial, to describe the process indicators (reach, dose, and fidelity) and impact (at the store and consumer levels) associated with each intervention, and to suggest potential next steps in research, practice, and policy.

## Methods

### Data sources

**Box. Search terms for systematic review of small-store interventions**

- corner store
- small food store
- retail food store
- bodega
- tienda
- store intervention
- store program
- store trial
- food retail [and] intervention
- food retail [and] program
- food retail [and] trial
- food environment [and] intervention
- food environment [and] program
- food environment [and] trial
- food access
- food availability
- food desert
- produce [and] availability
- produce [and] access
- fruit [and] vegetable [and] access
- fruit [and] vegetable [and] availability

From May 2009 through September 2010, we searched the peer-reviewed literature and "gray" literature. Only literature after 1990 was considered. Gray literature included newsletters, published (non-eer reviewed) articles, policy briefs or reports, published trial materials, and conference presentations. Using fixed search terms, we first conducted a PubMed search of peer-reviewed literature to identify small-store intervention trials designed to improve access to healthy foods. We then posted requests on the Healthy Corner Store Network (HCSN) listserv, conducted HCSN website searches, reviewed the abstracts from nutrition and food policy conferences, and consulted with colleagues. We performed searches using the same methods and fixed search terms every 6 months during the review period (Box). We looked for trials conducted in the United States and abroad.

Small stores were defined as having fewer than 10 employees and less than 1,000 square feet of floor space. Corner stores were urban small stores that were independently owned. Convenience stores were small retailers that were part of national or regional chains. Gas station stores were retail stores for servicing motor vehicles that also carried a limited selection of foods and beverages. Bodegas or tiendas were Hispanic-owned small ethnic-food stores. Urban areas were defined as census block groups with a total population of at least 2,500 and an overall density of at least 500 people per square mile. Rural areas were all territory outside urban areas.

### Trial selection

We initially identified 28 trials; 8 were drawn from PubMed. All identified food-store trials were reviewed for inclusion using the following criteria: 1) a focus on small food stores (although other food sources such as supermarkets and restaurants could be part of the study), 2) a completed impact evaluation (eg, pre-post assessment, use of a comparison group, exposure assessment), and 3) some form of written documentation (eg, peer-reviewed journal article, newsletter, other published article, policy brief or report, published trial materials, or conference presentation) that included a description of all implemented intervention and evaluation strategies and is written in English. Sixteen trials met inclusion criteria.

To mitigate bias, we documented the search process and the decisions that were made for each trial document. Two primary reviewers (P.G., M.R.), working independently, screened and selected trials. Each eligible trial was systematically appraised in terms of study design, interventions, outcome measures, fidelity of the implementation of the interventions, and trial findings. Disagreements were adjudicated by a secondary independent reviewer (J.G.).

### Data extraction and analysis

The 2 primary reviewers independently extracted and analyzed data by carefully reviewing all documents. The secondary reviewer developed the system of extracting data and coding variables. Variables, such as store type, were based on industry definitions. The 2 primary reviewers conducted interrater reliability assessments to assure consistency in coding. The secondary reviewer resolved discrepancies noted by the 2 primary reviewers and identified and adjudicated other discrepancies that might affect reliability and analysis.

Primary reviewers were instructed to extract data for each variable and to organize data using a trial as the unit of analysis. The data, which were summarized in 3 tables, were descriptive and comprehensive. These tables were submitted via e-mail to all trial managers (n = 16) for review and revision. Six months later, 11 of the 16 trial managers participated in semistructured phone interviews, which were designed to supplement and verify information on trial components, evaluations, and results. The remaining 5 trial managers did not respond to our request for an interview or were no longer involved with the trial.

After the initial review and follow-up, we created categories and terminology to provide comparability between extracted data. Primary reviewers extracted and reported data in accordance with this predetermined structure. The tables were modified accordingly. The secondary reviewer confirmed data accuracy using initial review findings, e-mail correspondences, interview transcripts, and extraction and reporting guidelines.

The analytic approach used to assess the trials was therefore based on the presence or absence of a standard set of quality criteria (eg, randomization, use of control groups) and the report of impact at the store and consumer levels. Meta-analytic techniques were not used, given the heterogeneity of outcome data, which did not permit the creation of summary estimates of impact.

## Results

Of the 16 trials (25-62) that met the inclusion criteria, 8 trials (25-42) provided peer-reviewed published materials. We therefore relied on gray literature for the remaining 8 evaluated trials (43-62).

### Target populations

Eleven trials focused on urban settings, and 5 focused on remote or rural populations. Four trials took place outside the United States (Tables 1a - 1c). All of the trials focused on low-income populations; most targeted racial/ethnic minority communities, including African Americans (n = 7), Hispanics (n = 6), American Indians/First Nations (n = 2), Pacific Islanders (n = 2), and Australian aboriginal peoples (n = 1).

### Behavior change theory

Thirteen trials explicitly mentioned theoretical frameworks that guided their design. Projects such as Vida Sana Hoy y Mafñana (61,62), the Healthy Food Retailer Inititive (50-52), and Baltimore Healthy Stores (27,28) most frequently used social cognitive theory (n = 7) and social ecological theory (n = 4). Other trials used community-based participatory research, a unique theory-of-change model, and environmental justice and sustainability models.

### Primary goals

Nine trials articulated their primary goal as improving access to healthy foods or, in some trials, fruits and vegetables (n = 4). Only 4 trials stated their primary goal as changing food purchasing and consumption patterns, but all 16 trials listed it as an indirect goal. Two trials, Vida Sana Hoy y Mafñana (61,62) and Baltimore Healthy Stores (27,28), mentioned changing store-owner attitudes as a primary goal. Three trials stated reducing risk for diet-related diseases as a long-term goal.

### Types of small food stores participating

Corner stores were the most frequently mentioned small-store types (n = 12). Less frequently mentioned were convenience stores (n = 3), bodegas/tiendas (n = 3), and liquor stores (n = 2). Examples of trials focusing on corner stores include the Live Well Colorado Corner Store Initiative (46,47) and Steps to a Healthier New Orleans Corner Store Initiative (57-60).

### Intervention strategies

**Promoted foods**

All 16 trials emphasized increased stocking of healthy foods, and 15 emphasized fresh produce promotion. Five trials focused exclusively on promoting produce. The other 11 trials, such as the Healthy Food Retailer Initiative (50-52) and Live Well Colorado Healthy Corner Store Initiative (46,47) also aimed to improve availability of other healthy foods, such as low-fat milk, whole-grain breads, reduced-fat snack foods, and canned vegetables. Five trials, Marshall Islands Healthy Stores (34,35), Healthy Foods Hawai'i (32), Apache Healthy Stores (25), Zhiwaapenewin Akino'maagewin (40-42), and Baltimore Healthy Stores (27,28), introduced healthy foods in phases (eg, snacks and beverages). Two trials sought to reduce the availability of unhealthy foods.

**Health promotion and communication**

Twelve trials used in-store signage (eg, shelf labels and posters) for point-of-purchase promotions. Seven trials, such as the Scottish Grocers Federation Healthy Living Neighborhood Shop (33), used media outside of the stores. Zhiwaapenewin Akino'maagewin (40-42) and Baltimore Healthy Stores (27,28) used educational flyers and promotional giveaways. Two trials, Apache Healthy Stores (25) and Healthy Bodegas (43-45), also used diverse multilingual social marketing materials in community venues (eg, newspapers). Three trials, including the Live Well Colorado Healthy Corner Store Initiative (46,47), used coupons or vouchers to increase healthy food purchases, and 7 trials used cooking demonstrations or taste tests to introduce unfamiliar healthy foods.

**Community engagement**

A common community engagement strategy (n = 8) was the use of stakeholder workshops to design and refine interventions. The South Los Angeles Healthy Eating, Active Communities trial (48,49) used community meetings as a forum to bring store owners and community members together to discuss intervention strategies (eg, store-front murals). The San Francisco Good Neighbors Program (36-39) worked to build relationships between government offices and community organizations.

**Store owner training**

Five trials worked directly with store owners and staff to provide general health education and business training (eg, stocking and handling fresh produce). Vida Sana Hoy y Mafñana (61,62) emphasized employee and manager capacity-building efforts. Baltimore Healthy Stores (27,28) provided healthy food stocking and cultural guidelines for Korean American small-store owners.

**Structural modifications**

Two trials worked to improve the small-store refrigeration system. One grocery store was stocked with a new energy-efficient refrigerator and used green materials to improve the store infrastructure (55,56). Another monitored refrigeration systems to ensure effective use (48,49). Three trials, including the Scottish Grocers Federation Healthy Living Neighborhood Shop project (33) and Vida Sana Hoy y Mafñana (61,62), emphasized stocking and providing display stands to sell fresh produce. Four trials moved unhealthy products to the back of the store and shifted healthier items closer to the point of purchase.

**Pricing**

Six trials included intervention strategies to reduce the cost of foods or products related to food procurement at the consumer or store level. Three trials, Baltimore Healthy Stores (27,28), Have a Heart Paisley (29-31), and Healthy Eating, Active Communities (48,49) provided coupons or vouchers for consumer purchases. Healthy Foods Hawai'i (32) and Baltimore Healthy Stores (27,28) provided cash incentives (ie, gift cards for use with their distributor or wholesaler) to store owners to purchase healthy foods. One trial, Live Well Colorado (46,47), provided store loans for business expansion and structural changes that would encourage the stocking and sale of healthy foods. Outback Stores (53,54) discounted healthy foods.

### Evaluation strategies

All 16 trials included pre- to post-intervention evaluations (Tables 2a - 2c). Only 6 trials included a comparison group. Five trials, Apache Healthy Stores (25), Healthy Foods Hawai'i (32), Vida Sana Hoy y Mafñana (61,62), Zhiwaapenewin Akino'maagewin (4-6), and Baltimore Healthy Stores (27,28), conducted randomized control trials, pre-post assessments, and exposure evaluations. Three trials, Have a Heart Paisley (29-31), Healthy Living Neighborhood Shop (40,41), and Marshall Islands Healthy Stores (34,35) assessed change using pre-post assessment and exposure evaluations. Seven trials, Healthy Bodegas (43-45), Live Well Colorado (46,47), Healthy Eating, Active Communities (48,49), Healthy Food Retailer Initiative (50-52), Outback Stores (53-54), Steps to a Healthier New Orleans (57-60), and the Good Neighbors Program (36-39), used pre-post assessment only. The Romano's Grocery Store Renovation (55,56) trial used a pre-post assessment and a policy analysis. Trials varied in terms of dependent variables (eg, change in produce stocking vs change in low-fat milk sales) and summary measures (eg, the use of means vs differences).

**Process measures**

Fifteen trials collected some form of process data, 14 of which collected both qualitative and quantitative data. Process data focused on availability of promoted foods, the presence of planned signage and other intervention materials, and store owner/manager engagement. The Zhiwaapenewin Akino'maagewin trial (40-42), Baltimore Healthy Stores trial (27,28), and Healthy Bodegas trial (43-45) also conducted store owner interviews to understand barriers to stocking.

**Store impact**

Fifteen trials assessed changes in availability of healthy foods; all used pre-post assessments. Ten assessments focused exclusively on perishable goods (produce, and, in 1 case, milk). Nine trials assessed impact on both food stocking and sales. Given the lack of owner-recorded sales data, the Baltimore Healthy Stores trial (27,28) conducted weekly store-owner recall evaluations. Eleven trials, including Baltimore Healthy Stores (27,28), also examined impact on the store owners'and managers' psychosocial variables, including food-related knowledge, intentions, and outcome expectations for stocking healthy foods.

**Consumer psychosocial impact**

Using pre-post assessments (n = 13), comparison group evaluations (n = 5), and exposure evaluations (n = 7), 14 trials (8 of which used multiple methods) examined impact on consumer psychosocial characteristics. Of these, the most frequently assessed outcomes were consumer food-related knowledge (n = 11), intentions (n = 9), and self-efficacy (n = 8). Less frequently assessed were attitudes about stocking healthier foods (n = 3), perceived barriers to healthy food purchasing (n = 1), and outcome expectations (n = 1).

**Consumer behavioral impact**

Food purchasing patterns (eg, frequency of purchase) were the most commonly assessed consumer behavioral change (n = 14). Thirteen trials used pre-post evaluations to assess changes in purchasing behaviors, 5 of which used a comparison group. Eight trials examined change in diet using pre-post assessments, 5 of which used a comparison group. A quantitative food frequency questionnaire served as the primary tool for assessments for those trials. Four trials, including Vida Sana Hoy y Mafñana (61,62), used surveys focused exclusively on intake of a subset of foods, such as produce.

**Consumer health outcomes**

Only 4 trials examined health outcomes, all of which focused exclusively on body mass index (BMI) change.

### Food store trial findings

**Process evaluation**

Food stocking and in-store promotional materials were placed and maintained with moderate to high fidelity across all trials (Tables 3a - 3c).

**Food availability**

Overall availability of promoted foods increased in all of the trials, yet some trials varied in food availability, such as certain low-fat snacks (eg, Baltimore Healthy Stores [27,28]). Trials did not report impact on the quantity of foods, but 5 trials that focused on produce availability did report an increased number of varieties (Zhiwaapenewin Akino'maagewin trials [40-42], the Apache Healthy Stores [25], Baltimore Healthy Stores [27,28], Steps to a Healthier New Orleans Corner Store Initiative [57-60], and Romano's Grocery Store Renovation [55,56]).

**Food sales**

Significant increases in sales of promoted foods were reported among all trials that collected sales data (Apache Healthy Stores [25], Baltimore Healthy Stores [27,28], the Good Neighbors Program [36-39], Scottish Grocers Federation Healthy Living Neighborhood Shop [33], and Have a Heart Paisley [29-31]). Trials that measured produce sales observed 25% to 50% increases. Postintervention maintenance data were measured by only 1 trial, Baltimore Healthy Stores (27,28), which demonstrated increases in stocking and sales of promoted foods 6 months post-intervention.

**Consumer psychosocial impact**

Consumer impact data were available (in both peer-reviewed and gray literature) for 10 trials. For 7 trials, consistent increases in food and health-related knowledge were observed; each of these trials included comparison groups. Other findings, which varied by trial, included increased recognition of the availability of healthy foods (Romano's Grocery Store Renovation [55,56]) and increased intention to buy healthy foods (Healthy Eating, Active Communities [48,49]). Except for 1 trial, none reported significant changes in self-efficacy.

**Consumer behavioral impact**

Of the 10 trials that reported impact on consumer purchasing and consumption behaviors, 9 observed significantly increased purchasing frequency of at least 1 promoted food. Seven of the 10 trials reported increased purchasing, by weight, of promoted foods, including fruits and vegetables, low-fat milk, high-fiber cereals, and water.

**Obesity impact**

No significant changes in BMI were reported by the 4 trials that assessed this outcome.

## Discussion

Our findings indicate consistent improvements across most of the trials in the availability and sale of healthy foods, the purchase and consumption of those foods, and consumer knowledge. Most of the trials that showed positive impact used multipronged strategies (food provision, infrastructure, and health communication) designed to improve both access to healthy foods (supply) and consumption of those foods (demand), thus demonstrating the need for combined environmental and behavioral approaches in small-store interventions.

Several studies have demonstrated that price reductions, through discounts, coupons, vouchers, and loans, can positively affect consumer demand for and consumption of healthy foods (22,63,64). Although all of the trials presented in this review sought to increase access to healthy foods by improving availability, only 6 sought to increase access by providing cost-related incentives. Research on increasing consumer demand for healthy foods by manipulating price is needed.

Limiting the availability of unhealthy food should also be considered. Four trials implicitly sought to discourage consumption by moving those products to the back of the store and shifting healthier items closer to the point of purchase. Only 2 aimed to reduce the availability of unhealthy foods. Three trials provided business training, which aimed to reduce profit loss associated with stocking and structural changes and was associated with improved healthy food availability. A combination of modifications to reduce unhealthy food stocking and consumption and training to reduce profit loss risks should be included in future trials and may be a sustainable policy-level approach. These modifications could be achieved through future mandates or licensing requirements for healthy food stocking.

Our systematic review indicated several deficiencies in small-store trials. Most trials assessed impact on store stocking of healthy foods, but many trials failed to consider sales data, and few examined impact on consumer outcomes, such as diet and health. No retail food-store trials have shown impact on health outcomes, such as obesity. The ability to influence health outcomes will require a more systematic evidenced-based approach to modifying the food environment, greater use of randomized controlled trials to evaluate program effectiveness (23), and publication in peer-reviewed literature to communicate findings.

Finally, efforts should be made to translate current small-store intervention findings into policy. Policies aimed at increasing healthy food availability have the potential to sustain improved nutrition among low-income populations (22-23). Such policies may need to account for increased food stamp or trial restrictions associated pwith the Special Supplemental Nutrition Program for Women, Infants, and Children (65), zoning or licensing mandates (66), economic incentives (coupons, produce coolers, tax breaks) (63,64), improved store facade or layout (63,64), and incentivized partnerships between producers, manufacturers, and distributors. Long-term multisectoral and multiagency efforts could address economic development in low-income areas with low food availability and high rates of obesity and chronic disease.

This systematic review has several limitations. Our findings are more descriptive than definitive. Because the trials varied widely, we did not conduct a meta-analysis with summary estimates, which would have provided a more comprehensive and precise statement of findings. We did not require that trials included in our review publish data in peer-reviewed journals. Although our conclusions were drawn largely from peer-reviewed literature, we found support for them in the gray literature, which we included in this study because of the dearth of information on small-store interventions in peer-reviewed literature. As a result, our analysis lacks information on assessment tools, and our impact analysis lacks summary estimates, *P* values, and data on consumer psychosocial and behavioral changes, and we cannot assess the relative impact of different intervention strategies. Consistent and comparable evaluation data are lacking for 2 reasons: 1) the field is new and emerging, and 2) many assessed trials were funded by small nonprofit organizations without the resources to publish in academic journals. These limitations underscore the need for standardized evaluation methods for and peer-reviewed articles on small-store interventions.

We provide the first systematic review of small-store interventions as a potential approach for addressing the obesity and diet-related chronic disease epidemics in the United States and internationally. Many of the findings presented are derived from gray literature, which may challenge their credibility. Nevertheless, the weight of the evidence supports the use of this approach to improve small-store stocks and sales of healthy foods, consumer psychosocial factors, and food purchasing and consumption behaviors. Further research is needed to determine the best combination of interventions for small-store trials.

## Figures and Tables

**Table 1a.** Description of Small-Store Intervention Trials 1-6

**Intervention Components**	Apache Healthy Stores (25)	Baltimore Healthy Stores (27,28)	Have a Heart Paisley – Changing Lifestyle (29-31)	Healthy Bodegas (43-45)	Live Well Colorado (46,47)	Healthy Eating, Active Communities (48,49)
**Data source**	Peer review article Website	Peer-reviewed article Printed materials	Peer-reviewed article Interview Website	Interview Conference presentation Website	Printed materials Interview	Printed materials Interview
**Target population**	San Carlos Apache American Indian Low-income	Baltimore African American Low-income Urban	Scotland Rural Low-income	New York African American/ Hispanic Low-incomeUrban	Denver African American/ Hispanic Low-incomeUrban	Los Angeles African American/ Hispanic Low-income Urban
**Model/theory**	Social cognitive theory	Social cognitive theory	Social cognitive/ learning theory	Social ecological model	Social ecological model	Theory of change
**Goal**	Availability Consumption Psychosocial	Availability Consumption Psychosocial	Affordability Consumption	Availability Affordability Consumption	Availability Consumption	Availability Affordability Consumption
**Food**	Produce Low-fat dairy Water Whole grain Healthy snacks	Produce Low-fat dairy Water Whole grain Healthy snacks	Produce	Water Low-fat dairy Whole grain Other	Snacks	Produce
**Intervention** **strategies**	Signage Shelf labels Handouts Giveaways Coupons Taste test Community promotion	Signage Shelf labels Handouts Giveaways Coupons Taste test Owner education	Signage Store owner Discounts Community promotion	Signage Shelving Store layout Owner education Supply Permits Community promotion	Community promotion Store owner Discounts Loans	Refrigeration Store layout Signage Handouts Coupons Community promotion

**Table 1b.** Description of Small-Store Intervention Trials 7-11

**Intervention Components**	Healthy Food Retailer Initiative (50-52)	Healthy Foods Hawai'i (32)	Healthy Living Neighborhood Shop (33)	Marshall Islands Healthy Stores (34,35)	Outback Stores (53,54)
**Data source**	Printed materials Interview Website	Peer review article Website	Peer review article Interview Website	Peer review article Website	Interview WebsiteOther
**Target population**	Hartford, Connecticut African American/HispanicLow-income	Honolulu, Hawai'i Pacific Islanders Low-income	Glasgow, Scotland Low-income	Republic of Marshall Islands Pacific Islanders Low-income	Australia Low-income Remote
**Model/theory**	Social ecological model	Social cognitive theory	Theory of reasoned action	Social cognitive theory	Not stated
**Goal**	Availability Consumption	Availability Consumption	Availability Consumption	Availability Consumption Psychosocial	Availability Affordability Consumption
**Food**	Produce Low-fat dairy Whole grain	Produce Low-fat dairy Water Whole grain	Produce	Produce Low-fat dairy WaterWhole grain	Snacks
**Intervention** **strategies**	Shelving Distribution Partnerships	Signage Shelf labels Handouts Giveaways Taste test Community promotion Store owner Discounts	Refrigeration Shelving Signage Store owner Education Produce lists	Signage Shelf labels Handouts Giveaways Taste test Community promotion	Store owner discounts Business training/loans Pricing Community promotion

**Table 1c.** Description of Small-Store Intervention Trials 8-16

**Intervention Components**	Romano's Grocery Store Renovation (55,56)	Steps to a Healthier New Orleans (57-60)	The Good Neighbors Program (36-39)	Vida Sana Hoy y Mañana (61,62)	Zhiwaapenewin Akino'maagewin (40-42)
**Data source**	Interview Program materials Other	Program materials Conference presentation	Peer-reviewed article Program materials Interview	Interviews Conference presentation Other	Peer-reviewed articles Website Other
**Target population**	Philadelphia African American/ Hispanic Low-income Urban	New Orleans African American Low-income Urban	San Francisco Low-income Urban	North Carolina Hispanic Low-income Urban/rural	Western Ontario First Nations Low-income
**Model/theory**	Social ecological model	Other	Environmental justice and sustainability model	Social cognitive theory Social ecological model	Social cognitive theory
**Goal**	Availability Consumption	Availability Consumption	Availability Affordability Consumption	Availability Affordability Consumption	Availability Consumption Psychosocial
**Food**	Produce Low-fat dairy Whole grain	Produce Low-fat Dairy Whole grain	Snacks	Produce	Produce Low-fat dairy Water Whole grain Snacks
**Intervention** **strategies**	Refrigeration Shelving Store layout Distribution Partnerships	Signage Community promotion	Store owner education Business training Distribution Partnerships Community promotion	Signage Business training Ready-to-eat produce bar	Signage Shelf labeling Handouts Giveaways Community promotion

**Table 2a.** Evaluation Strategies of Small-Store Intervention Trials 1-6

**Strategy**	Apache Healthy Stores (25)	Baltimore Healthy Stores (27,28)	Have a Heart Paisley – Changing Lifestyle (29-31)	Healthy Bodegas (43-45)	Live Well Colorado (46,47)	Healthy Eating, Active Communities (48,49)
**Overall study design**	Pre-post assessment Comparison group – delayed intervention Exposure assessment	Pre-post assessment Comparison group – delayed intervention Exposure assessment	Pre-post assessment Exposure assessment	Pre-post assessment	Pre-post assessment	Pre-post assessment
**Feasibility and process measures**	In-depth interviews Process indicators (reach, dose, fidelity) Interventionist logs	In-depth interviews Direct observation – inventory Process indicators (reach, dose, fidelity) – logs	Semi-structured interviews Direct observation – inventory Process indicators (reach, dose, fidelity)	In-depth interviews Direct observation – inventory Process indicators (reach, dose, fidelity)	In-depth interviews Direct observation – inventory	In-depth interviews Focus group Process indicators (reach, dose, fidelity)
**Store impact measures**	Availability Sales Psychosocial (outcome expectations, intentions, self-efficacy to stock)	Availability Sales Psychosocial (outcome expectations, intentions, self-efficacy)	Availability Sales Food quality Psychosocial (intentions – voucher use)	Availability Sales Psychosocial (intentions to sell) Store layout Marketing (signage, shelf labels, coupons)	Availability Sales Marketing (signage, shelf labels, coupons)	Availability Sales Psychosocial (intentions to stock)
**Consumer psychosocial measures**	Knowledge Self-efficacy Intentions	Knowledge Self-efficacy Intentions	Knowledge Self-efficacy Intentions	Knowledge Attitude	None reported	Knowledge
**Consumer behavioral measures**	Purchasing Preparation Diet	Purchasing Preparation Diet	Purchasing	Purchasing	Purchasing	Purchasing Preparation Diet Label reading

**Table 2b.** Evaluation Strategies of Small-Store Intervention Trials 7-11

**Strategy**	Healthy Food Retailer Initiative (50-52)	Healthy Foods Hawai'i (32)	Healthy Living Neighborhood Shop (33)	Marshall Islands Healthy Stores (34,35)	Outback Stores (53,54)
**Overall study design**	Pre-post assessment	Pre-post assessment Comparison group Exposure assessment	Pre-post assessment Exposure assessment	Pre-post assessment Exposure assessment	Pre-post assessment
**Feasibility and process measures**	None collected	In-depth interviews Direct observation – inventory Process indicators (reach, dose, fidelity) – interventionist logs	Semistructured interviews Direct observation – inventory Process indicators (reach, dose, fidelity) – project diary	In-depth interviews Direct observation – inventory Process indicators (reach, dose, fidelity) – interventionist logs	None collected
**Store impact measures**	Availability (% junk vs healthy food)	Availability Sales Psychosocial (outcome expectations, intentions, self-efficacy)	Availability Sales Food quality Psychosocial	None collected	Availability Sales Food quality
**Consumer psychosocial measures**	None collected	Knowledge Self-efficacy Intentions Perceptions of cost, convenience	Knowledge Self-efficacy Intentions	Knowledge Self-efficacy Intentions	Knowledge Intentions
**Consumer behavioral measures**	None collected	Purchasing Diet Body mass index	Purchasing	Purchasing Preparation	Diet

**Table 2c.** Evaluation Strategies of Small-Store Intervention Trials 12-16

**Strategy**	Romano's Grocery Store Renovation (55,56)	Steps to a Healthier New Orleans (57-60)	The Good Neighbors Program (36-39)	Vida Sana Hoy y Mañana (61,62)	Zhiwaapenewin Akino'maagewin (40-42)
**Overall study design**	Pre-post assessment Policy analysis	Pre-post assessment Comparison group exposure assessment	Pre-post Assessment	Pre-post assessment Comparison group – delayed intervention Exposure assessment	Pre-post assessment Comparison group – delayed intervention Exposure assessment
**Feasibility and process measures**	In-Depth interviews	In-depth interviews Direct observation – inventory	In-depth interviews Process indicators (fidelity)	In-depth interviews Direct observation- inventory Process indicators (reach, dose, fidelity)	In-depth interviews Direct observation – inventory Process indicators – interventionist/teacher logs
**Store impact measures**	Availability Sales Food quality Marketing	Sales records from 2/20 stores	Availability Sales Food quality Store layout Psychosocial	Availability	Availability Sales Food quality
**Consumer psychosocial measures**	Attitude	None collected	Attitude	Knowledge Self-efficacy Intentions Outcome Expectations	Knowledge Intentions
**Consumer behavioral measures**	Purchasing	None collected	Purchasing Diet	Purchasing Diet	Diet

**Table 3a.** Results of Small-Store Intervention Trials 1-6

**Results**	Apache Healthy Stores (25)	Baltimore Healthy Stores (27,28)	Have a Heart Paisley – Changing Lifestyle (29-31)	Healthy Bodegas (43-45)	Live Well Colorado (46,47)	Healthy Eating, Active Communities (48,49)
**Feasibility and process**	Store: High dose, high reach, medium/high fidelity Community: medium/high fidelity Individual: high dose, high reach	Interactive sessions*:* high dose, high reach Owner education: medium/high dose, medium/high fidelity Availability/ marketing: medium/high fidelity	Coupons*:* high reach, high dose, medium/high fidelity Marketing: high dose, high reach, high fidelity	Owner education: high fidelity Signage*:* high dose, high fidelity	Marketing/community promotion*:* high fidelity	Shelf labeling
**Store impact**	Increased sales (intervention vs comparison)	Increased availability Increased sales (sustained 6-months post-intervention)Increased Self-efficacy	Increased availability Increased sales (correlated w/ coupons)Increased coupon use	Increased availability (low-fat dairy) Increased sales (low-fat dairy)	Increased sales (produce) Increased knowledge (store owner) Improved produce storage	Increased availability (produce) Improved produce storage Increased customers
**Consumer psychosocial impact**	Increased knowledge	Increased intentions	Increased knowledge Perceived benefits	Not available	Not available	Increased knowledge Increased intentions
**Consumer behavioral impact**	Increased purchasing Increased consumption – promoted foods Decreased consumption – unhealthy alternatives	Increased purchasing (correlated with shelf labels) Increased prep	Increased purchasing (frequency, volume, variety) Increased consumption	Not available	Not available	Increased purchasing Increased consumption

**Table 3b.** Results of Small-Store Intervention Trials 7-11

**Results**	Healthy Food Retailer Initiative (50-52)	Healthy Foods Hawai'i (32)	Healthy Living Neighborhood Shop (33)	Marshall Islands Healthy Stores (34,35)	**Outback Stores (53,54)**
**Feasibility and process**	Not collected	Overall: medium dose, reach, and fidelity Individual and store: high dose, reach, and fidelity	Produce quality, availability: high fidelity Produce delivery: high delivery and reach Shelf labeling/ marketing/shelving: high fidelity	Overall: medium dose and reach, high fidelity	Management compliance: high fidelity Recruitment of indigenous employees: high fidelity
**Store impact**	Increased availability: produce Decreased availability: unhealthy snacks	Not collected	Increased sales: produce (correlated with marketing)	Not collected	Increased availability and variety Decreased prices Increased turnover and gross profit
**Consumer psychosocial impact**	Not available	Increased knowledge	Increased knowledge: health benefits	Increased knowledge: diabetes, label reading	Not collected
**Consumer behavioral impact**	Not available	Increased purchasing Increased consumption: water, fiber	Increased purchasing: produce Increased consumption: produce	Increased purchasing and preparation	Not collected

**Table 3c.** Results of Small-Store Intervention Trials 12-16

**Results**	Romano's Grocery Store Renovation (55,56)	Steps to a Healthier New Orleans (57-60)	The Good Neighbors Program (36-39)	Vida Sana Hoy y Mañana (61,62)	Zhiwaapenewin Akino'maagewin (40-42)
**Feasibility and process**	Not available	Marketing: high fidelity and dose	Nutrition education, cooking demonstration, cookbook: high dose and fidelity	Employee training: medium to high fidelity ProduceEquipment: medium to high fidelity Marketing: high fidelity Stocking: high fidelity	Schools/store: medium reach and fidelity Community: high dose and reach
**Store impact**	Increased availability: produce Decreased prices	Increased availability: produce, fiber, low-fat dairy	Increased sale: produce Decreased sales: alcohol/tobacco	Increased availability: produce (post-intervention) Decreased availability: produce (at follow-up)	Not collected
**Consumer psychosocial impact**	Increased knowledge: healthy food identification	Not collected	Not available	Decreased self-efficacy	Increased knowledge: healthy food identification
**Consumer behavioral impact**	Increased purchasing Increased consumption Increased consumers	Not collected	Not available	Increased consumption: produce	Increased purchasing
